# Decision-Based Epistemology: sketching a systematic framework of Feyerabend’s metaphilosophy

**DOI:** 10.1007/s11229-020-02934-3

**Published:** 2020-11-27

**Authors:** Daniel Kuby

**Affiliations:** Fachbereich Philosophie, Universität Konstanz, Constance, Germany

**Keywords:** Karl Popper, Viktor Kraft, Scientific philosophy, Paul Feyerabend, Metaphilosophy, Methodological rules, Axiology, Epistemic voluntarism, Realism

## Abstract

In this paper I defend the claim that Paul Feyerabend held a robust metaphilosophical position for most of his philosophical career. This position I call Decision-Based Epistemology and reconstruct it in terms of three key components: (1) a form of epistemic voluntarism concerning the justification of philosophical positions and (2) a behaviorist account of philosophical beliefs, which allows him (3) to cast normative arguments concerning philosophical beliefs in scientific methodology, such as realism, in terms of means-ends relations. I then introduce non-naturalist and naturalist variants of his conception of normativity, which I trace back to his mentors Viktor Kraft and Karl Popper, respectively. This distinction, introduced on the metaphilosophical level, can can be put to use to explain key changes in Feyerabend’s philosophical proposals, such as the viability of his methodological argument for realism. I conclude that this Decision-Based Epistemology should be further explored by historically embedding Feyerabend’s metaphilosophy in a voluntarist tradition of scientific philosophy.

## Introduction

In this paper I am going to defend the claim that Paul Feyerabend held a robust metaphilosophical position, i.e. a structured set of beliefs about how to conceive of philosophy. This metaphilosophical position, which I call his Decision-Based Epistemology, can be reconstructed in terms of a structured set of beliefs about how knowledge in general and, specifically, scientific knowledge can be conceived and what follows from this in terms of what doing philosophy (of science) can mean. Indeed, my claim is that this is the metaphilosophical position from which Feyerabend addressed epistemological matters for most of his intellectual life. At its core, this conception of philosophy rests on a form of epistemic voluntarism, according to which philosophical beliefs are not justified by evidence, but by an appeal to epistemic values; and it uses a behaviorist account of philosophical beliefs, which makes these beliefs amenable to pragmatic warrant. Arguments for a certain philosophical claim are thereby deployed as means-ends arguments. Feyerabend used the methodology of science as the central arena in which these philosophical arguments can be put to work; and developed a domain of axiological reasoning, as the normative grounding of his position, which can be either dependent or independent of scientific practice.

In advancing this account, I question Eric Oberheim’s influential (and in many ways excellent) interpretation of Feyerabend’s philosophy (Oberheim 2006). In spite of the “received view that Feyerabend simply had no coherent overall philosophy”, Oberheim correctly argues that Feyerabend had a “coherent, positive conception of philosophy that he developed in the 1950s, and practiced through to the early 1990s” (Oberheim 2006, p. vii). Someone’s “conception” is customarily spelled out as a set of philosophical claims forming a philosophical position, i.e. a set of beliefs to which a person qua philosopher is committed to. However, the main thesis of Oberheim’s interpretation claims that Feyerabend did not have a philosophical conception in this sense. Instead, he argues that

Feyerabend was like a chameleon, adapting to the philosophical landscape as it underwent dramatic (perhaps even revolutionary) changes over four decades. As his career progressed, Feyerabend increasingly emphasized his pluralistic philosophical method. He was more and more explicit about his strategy of temporarily adopted conflicting philosophical standpoints, and using and developing ideas that are inconsistent with each other for the purpose of criticizing existing points of view (Oberheim 2006, p. 270).

Let’s call this Oberheim’s “lack of commitment” thesis, according to which Feyerabend’s conception of philosophy purposely didn’t commit him to any philosophical view. The positive side of Feyerabend’s philosophy, then, is not to be found in a set of beliefs he was committed to, but in his pluralist method and aim, which he incessantly pursued through his philosophical career. What becomes clear in this exposition is that, while he is denying the existence of a philosophical position, Oberheim is ascribing to Feyerabend a *metaphilosophical* stance, i.e. a way of conceiving and doing philosophy.^1^

On the one side, I agree with Oberheim’s claim that Feyerabend adopted a quite stable metaphilosophical stance throughout his career—*pace* Preston (1997, p. 7), who claims the existence of a strong discontinuity between the early and later Feyerabend.^2^ On the other side, on my view this stance can and should be explicated—*pace* Oberheim—in terms of a position as customarily understood, i.e. a set of philosophical beliefs to which a person qua philosopher is committed to. This is where I diverge from Oberheim to present my own reconstruction of just what these beliefs are.

Interestingly, Feyerabend didn’t expose much of these beliefs in his writings; they survive mostly in scattered remarks, with one important exception (see his 1962, discussed below); on the few occasions he explicitly addressed his beliefs, he simply stated them—looking out for any real justificatory talk, we come home almost empty-handed. Yet Feyerabend’s scarce and self-evidential manner of presentation shouldn’t be interpreted as a dogmatic trait. Instead, the mode of presentation should be taken as an indicator of the obviousness—at least to Feyerabend—of the beliefs presented. The Decision-Based Epistemology here sketched forms a background to and a framework for his philosophical work proper. These background beliefs are not the beliefs he challenged or justified, they are the “hinge” beliefs that enabled him to challenge and justify further beliefs.

Proposing the framework of Decision-Based Epistemology as an account of Feyerabend’s conception of philosophy has interesting consequences, two of which I will discuss in this paper. First, these metaphilosophical views tie him to a specific (but not homogeneous) philosophical heritage, namely, Viennese scientific philosophy (Stadler 2010). Feyerabend acquired these views early on. They are a result of his philosophical apprenticeship in pre- and post-war Vienna (Kuby 2010). This Decision-Based Epistemology, then, allows us to see Feyerabend as a token of a much larger development, namely a *voluntarist tradition* within scientific philosophy. Proponents of this tradition highlight in several ways the role of volitional decisions in science and its philosophy.^3^ In the case of the Berlin and Vienna Circles, this viewpoint opposes long held wisdom about Logical Empiricism, for both the “logical” and the “empiricist” components have been viewed as the antithesis of discretionary choice in their account of what knowledge is. Rejecting this view and building on work by Carus (2007) and Richardson (2011), I contend that most logical empiricists actually embraced a discretionary view of knowledge by rejecting a compulsory conception of rationality and belief and by adopting a permissive conception typical of epistemic voluntarism. In this paper I will sketch one part of this connection by weaving into my story two of Feyerabend’s teachers, during his formative years and his early career: Viktor Kraft and Karl Popper.

Second, these metaphilosophical beliefs are remarkably stable through Feyerabend’s intellectual life. They form the basic continuity within Feyerabend’s philosophy, yet have a peculiar characteristic. I claim that slight variations, adjustments or changes at the metaphilosophical level can have (and did have) major repercussions at the philosophical level. One of the features of using Decision-Based Epistemology as a framework for Feyerabend’s metaphilosophy is that it is possible to explain a particular instance of a major change at the philosophical level by means of quite circumscribed changes at the metaphilosophical level. A case in point is Feyerabend’s advancement (and subsequent abandonment) of his methodological argument for realism, which will be presented in this paper.^4^

The plan for this paper is as follows: In Sect. 2, I first sketch the main components of Feyerabend’s Decision-Based Epistemology: a form of epistemic voluntarism and a behaviorist account of philosophical beliefs, leading to the deployment of arguments about philosophical beliefs, such as realism, on pragmatic grounds in the context of scientific methodology. In Sect. 3, I introduce the difference between naturalist and non-naturalist conceptions of normativity of methodological rules and show how this distinction can be used to track the development of this metaphilosophical framework, focusing on the philosophical consequences of the non-naturalist conception in the late 1950s to the mid 1960s, namely, a substantive axiological reasoning connecting epistemic values with moral values. In Sect. 4, I show how naturalist and non-naturalist variants of the normativity of methodological rules can be found, respectively, in the early works of Feyerabend’s mentors Viktor Kraft and Karl Popper. I then argue how their accounts lead to a more “conservative” conception of axiological inquiry, on Kraft’s side, and to a more “liberal” one, on Popper’s side—a difference which can be mapped on Feyerabend’s developing conception of philosophy as well. Section 5 concludes by reaffirming the continuity of Feyerabend’s axiological reasoning in the context of Decision-Based Epistemology over his career and presents some outstanding questions.

## Decision-based epistemology

In this section I am going to give an overview of the main components of Feyerabend’s Decision-Based Epistemology (DBE). At its core, Feyerabend’s conception of philosophy rests on a form of *epistemic voluntarism*, according to which philosophical beliefs are not motivated by truth-conducive considerations, but by an appeal to epistemic values (2.1) and a *behaviorist account of philosophical beliefs* (such as realism), which makes them amenable to pragmatic warrant (2.2).

Then I introduce a distinction between *methodology*, as the central arena in which Feyerabend deployed philosophical arguments on pragmatic grounds; and *axiology*, as the normative grounding of Feyerabend’s discourse (2.3). This is a distinction that Feyerabend himself never made. All that I discuss under the headings of ‘methodology’ and ‘axiology’, Feyerabend discussed under the single heading of ‘methodology’. Thus Feyerabend speaks of “methodological decisions” where we want to distinguish between the methodological component (relating means to ends) and the axiological component (warranting aims as values). I introduce this distinction in order to offer a more fine-grained reconstruction of Feyerabend’s metaphilosophy.

Importantly, Feyerabend’s pragmatic reasoning raises the issue of *realizability* of epistemic aims through methodological rules, which becomes both a constraining notion for the choice of epistemic values and a warranting notion for methodological arguments (2.4).

Finally, the methodological argument for a *realistic interpretation of scientific theories* (or “realism” for short) is introduced as an example of Feyerabend’s DBE at work (2.5).

### Epistemic voluntarism

The backbone of Feyerabend’s metaphilosophy is a form of epistemic voluntarism.^5^ According to this view,

the procedure leading to the adoption of a philosophical position cannot be *proof* (proof shows that no other position could possibly be realized), but must be a decision on the basis of preferences. [...] These positions admit of a genuine choice. Philosophers have habitually judged the situation in a very different manner. For them, only one of the many existing positions was true and, therefore, possible. This attitude, of course, considerably restricts the domain of responsible choice. (Feyerabend 1965, p. 146, fn. 5).

Feyerabend rejects the customary notion that philosophical positions are truth-apt. Instead, he seems to hint at the view that philosophical positions can be understood as stances, to use van Fraassen (2002)’s terminology. Indeed, quite similarly to contemporary voluntarist positions, Feyerabend’s voluntarism is liberal in kind. It rejects a notion of compulsory belief and introduces a permissive one, involving choice, deliberation and decision. The only substantive constraint is given by the notion of “realizability”, which previews Feyerabend’s practical understanding of the nature of epistemology:

One should notice that [the rejection of the stability thesis] is also grounded in the methodological decision to incorporate only refutable statements into science (or into our knowledge generally). (According to my view, this is an entirely general property of epistemological problems. They are not solved by proofs, but by decisions, as well as by the (empirical or logical) evidence that the decisions made are realizable [...]) (Feyerabend 1999, p. 42; cf. Feyerabend 1960a, pp. 64–65).

It is difficult to see at first how this voluntarist conception does without the (more controversial) assumption of doxastic voluntarism, i.e. the thesis that belief-formation is subject to deliberation.^6^ Feyerabend’s talk of “procedure” and “adoption of a philosophical position” can indeed be read this way. To reduce the problematic import of this issue for my reconstruction,^7^ we have to keep in mind that Feyerabend’s view is limited to reflective beliefs that ideally result from a critical attitude, in which belief-formation is supposed to be the result of reflection. Furthermore, the way Feyerabend writes about and argues for philosophical views makes it quite clear that this voluntarist view is chiefly concerned with belief-justification. Here Feyerabend seems to presuppose that philosophical positions are quite generally not warranted on epistemic (in particular: evidential) grounds, but by appeal to pragmatic reasons. Since from this vantage point philosophical views are not truth-apt, the warrant of philosophical positions does not concern truth-makers, but is indirectly provided by appeal to “preferences” (Feyerabend 1965, p. 146, fn. 5). As we will see, for Feyerabend, such preferences are actually expression of substantive moral values.

### Behavioristic strategy

To make sense of the interplay between the epistemic and the pragmatic we have to introduce a further basic element of Feyerabend’s metaphilosophy, which he also acquired during his formative years: a radical behaviorist strategy applied to philosophical problems.^8^ Due to this strategy, central concepts in the philosophical tradition (such as “observation” and “realism”) are deflated and modeled in terms of observable behavioral patterns of an epistemic agent or a group of agents (e.g. scientists).^9^ This move is quite radical: philosophical concepts with long historical pedigrees are ‘demoted’ to public, observable dispositions.^10^ This way the treatment of philosophical concepts, including the adoption and rejection of beliefs about them, becomes susceptible to pragmatic warrant.

Let us introduce a central example, namely, the issue of realism about scientific theories; in particular semantic realism, the thesis that our best scientific theories should be interpreted literally, i.e. that their claims about theoretical entities are truth-apt.^11^ This thesis is usually understood to be upheld by evidential reasons that make semantic realism more likely to be true. But much of Feyerabend’s positive arguments for a realistic interpretation of scientific theories concern the question whether beliefs involved in semantic realism are *useful*, i.e. conducive to some further aim.^12^ Here the behaviorist strategy comes to the rescue: The argument doesn’t target the belief qua propositional attitude, but the behavioral patterns that are correlated with such a belief. They target how scientists *handle* scientific theories. To use van Fraassen’s voluntarist terminology, Feyerabend’s pragmatic arguments target philosophical stances, which are not believed on evidential grounds, but adopted, held and realized by human action.^13^

### Methodology

In Feyerabend’s ‘epistemic voluntarism’ quotation we encountered a specific proposition, namely “to allow only refutable statements into science”. This is a classic *methodological rule*. And indeed methodology is Feyerabend’s philosophical project that contains the kind of philosophical discourse we are to reconstruct: it bears the central arena in which he put his practical reasoning on display. True to a long philosophical tradition, this practical reasoning concerns means-ends relations. Laudan (1987) gives a clear exposition of the view, put to use here, that methodological rules consists of (often implicit) reasoning about means-ends relations:

Methodological rules do not emerge in a vacuum, and without context. Methodological rules or maxims are propounded for a particular reason, specifically because it is believed that following the rule in question will promote certain cognitive ends which one holds dear. By formulating methodological rules without reference to the axiological context which gives them their bite [...], one is systematically disguising the route to their warrant. I submit that all methodological rules should be construed not [...] as if they were categorical imperatives, but rather as hypothetical imperatives. Specifically, I believe that methodological rules, when freed from the elliptical form in which they are often formulated, take the form of hypothetical imperatives whose antecedent is a statement about aims or goals, and whose consequent is the elliptical expression of the mandated action (Laudan 1987, p. 24).

My interest here is not with the validity of Laudan’s claim as a general thesis about the nature of methodological rules, but as a view of methodological rules that perfectly mirrors Feyerabend’s. I apply Laudan’s claim as an interpretative one: In Feyerabend’s case, methodological rules are best understood as hypothetical imperatives, connecting cherished epistemic aims to effective means through which the aims can be realized (‘If one’s goal is *x*, one should do *y*’).^14^

So, in the example above, the methodological decision to allow only refutable statements into science is an epistemic norm introduced on the basis of the epistemic value of falsifiability. In order to have any bite, the instrumental relation will connect epistemic values (set as epistemic aims) to actionable means, cast as behavioural guidelines. At this juncture, epistemology meets action theory and the behaviorist strategy outlined above comes to the rescue. The explicit form of methodological rules is thus:

1. If agent *A* want to realize the epistemic aim *N* (and thus promote the epistemic value *V*), *A* should do *G*.

For the present discussion, the form (1) of methodological rules is an explication of what is meant by a *normative* conception of philosophy of science. Insofar as philosophy of science is concerned with the construction of methodological rules, philosophy of science has a normative task.^15^

### Realizability

Methodological rules, in either their implicit or explicit form, seem to be far removed from testable claims, having the form of (conditional) demands that epistemic agents are required to follow (if they want to achieve a certain epistemic aim). And the focus of this paper, starting in Sect. 3 (see especially Sect. 3.3), is on two mutually excluding ways in which the stipulation of the epistemic aim can be justified. But, importantly, the practical dimension of Feyerabend’s reasoning also raises the issue of the realizability of epistemic aims through methodological rules, which turns out to act as both a constraining notion on the choice of epistemic values and as a warranting notion for methodological arguments.

To show this, I will put to use Laudan’s view that

[methodological] rules, even if they do not yet appear to be truth-value bearing statements themselves, nonetheless depend for their warrant on the truth of such statements. [...] I am suggesting that we conceive rules or maxims as resting on claims about the empirical world, claims to be assayed in precisely the same ways in which we test other empirical theories (Laudan 1987, p. 24).

Indeed, the hypothetical imperative rests on an empirical connection between the means and the ends: the means have to bring about the ends, i.e.

(2) Doing *G* brings about *N*.

As in the previous subsection, I am not concerned with the validity of this further claim qua general philosophical thesis.^16^ Rather, I argue that Laudan’s claim matches quite nicely the argumentative use that I attribute to Feyerabend.^17^

It is important to note that the axiological component (i.e. the determination of which epistemic values to pursue) and the action-theoretic component of practical reasoning (i.e. behavioral guidelines set to realize these epistemic values) are not only supplemented by an empirical component; the very viability of specific conditional arguments modeled after (1) rests on the truth of contingent statements of the form (2). This allows us to appreciate Feyerabend’s additional clause that epistemological problems are not solved “by decisions” alone, but also “by the (empirical or logical) evidence that the decisions made are realizable” (Feyerabend 1999, p. 42; cf. Feyerabend 1960a, p. 65).

Indeed, the whole construction hinges on the notion of realizability. While aims are set by deliberation, they are not yet actionable goals; they become so if embedded in a practical (action-theoretic) context supported by empirical knowledge. If realizability was initially introduced as a minimal constraint on the voluntarist conception, an avoidance of “self-sabotage by one’s own lights” (van Fraassen 2004, p. 184), we see that an action-theoretic interpretation of the notion of realizability makes it part of the deliberation process.^18^ Just as we thought that evidential reasons were no longer in Feyerabend’s purview, evidence prominently reenters the game of warrant. This constraint on epistemic values (only those epistemic values which can be realized are *bona fide* candidates for the stipulation of epistemic aims^19^) and the justificatory pressure put on methodological rules (they must realize the stipulated epistemic values) looms constantly in the background of Feyerabend’s pragmatic reasoning. Yet, as I will detail in Sect. 3, the importance of realizability considerations varied widely over time in Feyerabend’s normative conception of philosophy of science.

### Decision-based epistemology at work: the methodological argument for realism

A notable instantiation of Feyerabend’s methodological reasoning, his methodological argument for realism, frames a realistic interpretation of scientific theories as a means to realize the epistemic goal of testability in the realm of science.^20^ Feyerabend’s methodological argument for realism builds on (1), where the antecedent contains valued epistemic norms and the consequent the prescription to interpret scientific theories realistically:

(3) If scientists want to increase the testability of scientific theories (and thus promote the epistemic value of falsifiability), they should interpret scientific theories realistically.

In the case of realism, the validity of the hypothetical imperative rests on a causal claim such as:

(4) Interpreting scientific theories realistically is more likely to maximize their testability than alternative interpretations.

Even if a hypothetical imperative like (3) is supported by (4), it is still a conditional argument. For a full argument we will want to reach a normative conclusion. I reconstruct Feyerabend’s methodological argument for realism as follows:

1. Theory-testing is a constitutive task of science. (Principle of Testability)
2. Interpreting scientific theories realistically is more likely to maximize their testability than alternative interpretations.
3. If scientists want to test scientific theories, they should interpret scientific theories realistically.
4. Scientists should interpret scientific theories realistically. (Realism)

This way of arguing for realism surely looks strange from the standpoint of the contemporary realism debate,^21^ so a few clarifications are in order. While today’s realism is discussed in terms of arguments that make a philosophical position more likely to be true, Feyerabend’s instrumental discussion of the epistemic aim of science frames realism as a conditional proposal connecting a means (the realistic interpretation of scientific theories) to an end (the testability of scientific theories). Furthermore, the content of Feyerabend’s realism, i.e. its restriction to the semantic dimension, looks far removed from scientific realism, given that contemporary empiricism does accept semantic realism as well.^22^

The reason for this, I submit, is historical and is easily explained by a shift in terminology. From Feyerabend’s vantage point, the methodological argument for realism is made to advance earlier *empiricism*, not scientific realism. Feyerabend’s terminology is rooted in the earlier logical-empiricist realism debate.^23^ In the first half of the 20th century a position like scientific realism (Psillos 1999) would have (and had) been rejected as untenable metaphysics by most empiricist parties involved. More precisely, the ontological and, in a more limited way, the epistemological components would have (and had been) opposed as “metaphysical”. The disagreement was about the semantic component. Proponents of a strict or relaxed verificationist approach would reject the semantic component (“positivism”), while a realist faction would accept in various ways the thesis that claims about theoretical entities could be truth-apt (“realism”). From the perspective of the later debate between scientific realism and constructive empiricism, the earlier discussion had been about the outlook of empiricism. In this limited context we can distinguish a realist strand—including Moritz Schlick, Karl Popper, Hans Reichenbach, Herbert Feigl and Viktor Kraft—to which Feyerabend’s methodological realism is an heir.^24^

I will use realism as a token for Feyerabend’s changing views on the philosophical level, rather than the metaphilosophical one, throughout the rest of this paper. Note in particular that to reach the conclusion we have to introduce a claim about science (Premise 1, or P1), here the Principle of Testability (PT). Already in one of the first appearances of the methodological argument for realism, Feyerabend understood PT to be a claim about the constitutive aim of science. As early as 1954 he argues for its constitutive nature by showing how it blocks (what is today known as) the Pessimistic Meta-Induction in scientific practice.^25^

While this stipulative understanding of the principle of testability remained constant, I will show that Feyerabend’s evolving attitude towards the nature of the principle, its justification as well as its status—indeed the nature, justification and status of any demand we could insert in P1—reflects Feyerabend’s prescriptive conception of philosophy of science with regard to science.

## Axiological reasoning between philosophy and science

In this section I make the case for the interpretational value of my proposal of a Decision-Based Epistemology as an account of Feyerabend’s metaphilosophy by showing how relatively circumscribed differences on the metaphilosophical level can explain momentous changes on the philosophical level. First, I introduce the difference between naturalist and non-naturalist conceptions of normativity relatively to the way in which the axiological determination of the epistemic aim in a methodological argument is addressed (3.1). I then proceed to sketch such changes in the chronological development of DBE from the late 1950s onwards (in which a non-naturalist conception prevailed) and in the second half of the 1960s (swinging back to a naturalist conception) (3.2). As an account detailing the connection between Feyerabend’s later naturalist conception of DBE and his adherence to the “historical turn” in philosophy of science is developed in Kuby (forthcoming), I will devote the rest of the section to give an account of Feyerabend’s earlier non-naturalist conception of DBE: the development of an axiological reasoning, connecting epistemic values and moral values, which is deemed independent from the axiological inquiry in the sciences (3.3) and led Feyerabend to uphold an interventionist conception of philosophy of science, i.e. the view that philosophy of science has standing to intervene in the development of science, not just to describe or analyze it (3.4).

### Naturalist and non-naturalist conceptions of normativity

In Sect. 2.4 I described how the notion of realizability works both as constraining and as warranting notion in the process of instrumental rationality. I also argued that it doesn’t change the basic situation of genuine choice in the deliberation process leading up to an epistemic stance. Now we have to ask: If goals give us a guide for choosing means, what can guide us to choose epistemic aims—and justify our choice? More concretely, what guides us in our choice of the epistemic aim of testability (PT) in the methodological realism argument?

I want to distinguish between two conceptions of normativity according to the way in which the axiological determination of the epistemic aim is addressed. On the one hand, a *naturalist conception of normativity*, according to which epistemic aims can be settled by reference to the axiological practice of science (this has been called “normative naturalism” by Laudan 1987, 1990).^26^ In this case, axiology falls back to methodology, as it were. On the other side, under a *non-naturalist conception of normativity*, epistemic aims cannot be settled by reference to the axiological practice of science. Therefore, the axiological discourse becomes independent of methodological considerations.

### The development of decision-based epistemology

In an initial phase, spanning over the first half of the 1950s, Feyerabend was willing to accept PT as a descriptive claim about the axiological practice of science, in line with the naturalist conception of normativity of his teacher Viktor Kraft. Thus I interpret Feyerabend’s early instantiations of PT, like “the scientist’s most important task: to correct scientific theories” (Feyerabend 2015, p. 23, amended translation; cf. Feyerabend 1954, p. 475), as a (putatively true) reference to a demand rooted in and justified by the axiological practice of science.^27^ Feyerabend has no problem simply referring (and deferring) to the norms of science in matters axiological.

In the second half of the 1950s Feyerabend showed increasing displeasure with such a loose naturalist construal of PT. Sustained by a growing non-naturalist sentiment, he came to view PT as an *independent* demand concerning the aim of science, not justifiable with reference to the axiological practice of science. Feyerabend refers to “naturalism” as used by Popper and he diligently attributes his own non-naturalist stance to Popper’s discussion and evaluation in Chapter V of *The Open Society and Its Enemies* (2012).^28^

Simply put, Feyerabend understands naturalism to be any doctrine that attempts to overlook the “dualism between nature and convention” (Feyerabend 1958a, p. 147). He thinks these attempts must fail in principle because they commit a basic naturalistic fallacy: “The simple logical point that decisions are never derivable from facts should show that in all its forms naturalism is based upon a logical fallacy” (Feyerabend 1960c, p. 337). But, as usual with Feyerabend, his substantive critique is consequentialist in kind. He interprets naturalism as a general attempt to attribute to nature features that are part of the domain of human volition (“nature” is understood to be any epistemic source arguably accessible by human beings yet not under the control of human volition). In this way, the man-made character of those features is obfuscated and deemed to be a part of the scaffolding of the world, of the way things are, unaffected by human choice and unchangeable by human intervention. Feyerabend’s effort to do justice to the man-made character of knowledge shows in his attempts to disclose the different ways in which human beings, scientists and philosophers in particular, dispense of choice.

To name only a few forms of naturalism which Feyerabend takes on: Choices can be placed out of reach in that they are disguised and objectified as supernatural enunciations, synthetic a priori truths, unchangeable common sense, facts of nature—and scientific dogma. Reference to the axiological practice of science, from a non-naturalist viewpoint so construed, hides the moment of choice, the volitional nature of the decisions involved.^29^ Also, his non-naturalist position was argued on the view that even after considering the constraints and reasons the notion of realizability has to offer, there remains a multiplicity of realizable, yet mutually exclusive epistemic aims. This is not an argument from conceivability. Feyerabend’s view on the history of knowledge is that various contrasting epistemic aims have been realized as a matter of fact (see Feyerabend 1962, discussed below).

In summary, neither reference to axiological practices of science nor realizability considerations can uniquely determine the aims of science. If they cannot, what can? Feyerabend’s own answer is that the structure of *decisions* can deliver reasons and constraints that empirical or practical considerations cannot. My interpretative claim, which I will develop in the next subsection, is that Feyerabend’s axiological conception consists of more formal considerations about ‘axiological consistency’ and ‘axiological hierarchy’ concerning relation between aims; as well as a strong substantive thesis about the relation between epistemology and ethics, in which moral values serve as a foundation for epistemic values.

In the first half of the 1960s Feyerabend exploited this version of DBE at length to argue for specific methodological aims, such as realism and theoretical pluralism, and against the more ‘descriptivist’ approaches of proponents of the historical turn, first and foremost Thomas Kuhn.^30^ This axiological reasoning, which peaked in a form of philosophical prescriptivism (see Sect. 3.4 below), eventually came under pressure from a seemingly unlikely source: Feyerabend’s specific interest in quantum mechanics. As argued in Kuby (forthcoming), his interest in the establishment of Bohr’s contribution to quantum theory led him to a re-appreciation of scientific practice as a serious testing arena of realizability claims (as sketched in Sect. 2.4), which would eventually undermine the general, non-contextual scope of his methodological arguments. The demise of general methodology initiated a cascade of consequences: It was for Feyerabend a reason to give up the belief that there is a rational justification that allows to inject epistemic demands into science from first (moral) principles; more than that, it meant that science can move justifiably along completely different lines, in that adopting morally objectionable epistemic values may be the right methodological move in a given scientific context. Science may not contribute to the well-being of mankind after all and justifiably so from the perspective of scientific progress. But then it is up to society to protect its moral demands—against scientific freedom if necessary. This conclusion was developed to extreme consequences in *Science in a free society* (1978).^31^

### Feyerabend’s axiological reasoning: the ethical foundations of epistemology

The only published work in which Feyerabend puts his non-naturalist conception of normativity on display is, to my knowledge, his Nellie Heldt lecture *Knowledge Without Foundations* (1962). In it, Feyerabend details his substantive axiological reasoning which is only alluded to in other writings. These lectures have also been called Feyerabend’s “most Popperian paper” (Preston 1997, p. 78) or “most explicitly Popperian work” (Collodel 2016, p. 41) not without reason.^32^ In them, he closely follows Popper’s account of the rise of a critical attitude and offers a passionate defense of it. The main topic is a discussion of two ideals of knowledge: Myth as a template for a form of knowledge in which certainty is sought and attained; and the rise of a critical attitude, in which knowledge is realized as a fallibilist enterprise. According to Feyerabend, these are not just different, but mutually exclusive ideals of epistemic values. Both epistemic values can be realized, as he shows by following a (quite Popperian) historical account of Presocratic philosophy.^33^ This leads to a situation in which a genuine choice between epistemic values presents itself and which can be only be resolved “on the basis of preferences”. How can preferences resolve such a choice? In this context, Feyerabend uses “preferences” to refer to substantive moral values. His surprising answer is that epistemic attitudes are embedded in much larger contexts, “ways of life”, as he calls them. For example, a preference for the ideal of fallible knowledge, which is expressed in the critical attitude in epistemology, is embedded in a *critical way of life*. Epistemic stances may seem like abstract options, but are not if the practical conditions of their realization are taken seriously. They are only live options if they are embedded in ways of life bound to “mould every activity of the human life, emotions and thoughts alike” (Feyerabend 1962, p. 58).^34^

If epistemic stances are embedded in ways of life, then the choice of an epistemic stance is, really, a choice for a certain form of life. Translated into axiology: epistemic values are part of a tightly interconnected web of human values. Let us call this Feyerabend’s *axiological holism*. This holism has two major consequences for Feyerabend’s axiological reasoning: First, the choice of epistemic values is highly constrained by ‘axiological consistency’ considerations: decisions in one domain of human life will affect decisions in other domains. Second, epistemic values are not only connected to other values, they are dependent on other values, leading to an ‘axiological hierarchy’. This allows Feyerabend to state a result that—as he readily acknowledges—“is very different indeed from what seems to be the commonly accepted point of view in epistemology”: The question of choice of epistemic values

leads at once to the following fundamental problem: which attitude shall we adopt and which kind of life shall we lead? This is the most fundamental problem of all epistemology. (Feyerabend 1962, p. 55)^35^

[W]e are confronted here with a real decision, that is, a real choice with a situation which has to be resolved on the basis of our demands and preferences, and which cannot be resolved by proof. It is easy to see that these demands and these preferences concern the welfare of human beings and are therefore ethical demands: epistemology, or the structure of knowledge we accept, is grounded upon an ethical decision (Feyerabend 1962, p. 56).

This is a strongly heterodox result indeed. I propose to use the concept of “entailed decision” (*Folgeentscheidung*), first introduced by Hans Reichenbach (1961), to capture the web of interrelated decision-making resulting from these axiological consistency and hierarchy considerations.^36^ Let’s call this Feyerabend’s strong view of *axiological entailment*, which makes such consequentialist arguments possible. Reichenbach, himself a protagonist of the voluntarist tradition in scientific philosophy, introduced the notion of “entailed decision” to counter the claim of arbitrariness associated with the concept of choice in epistemology and to highlight the constraints put on the voluntarist deliberation process (Reichenbach 1961, p. 15). What was outside of Reichenbach’s purview is Feyerabend’s novel claim that epistemological decisions are determined by ethical decisions, connecting moral values to epistemic values.^37^

Feyerabend’s axiological conception thus provides an answer concerning how to go about choosing epistemic values—and how to justify this choice. It does so by advancing a thesis about a strong entailment between ethical and epistemological decisions, involving respectively moral and epistemic values: “I have shown that the choice between these two forms of life is a genuine choice which must be made individually by everybody who is presented with it on the basis of his own demands and his own ideas” (Feyerabend 1962, p. 71).

In the case of his argument for realism, the epistemic value of testability (stipulated by PT) is entailed by valuing the critical tradition in the pursuit to realize a critical way of life. Importantly, Feyerabend doesn’t offer any suggestion as to why one should value a critical way of life above others—in accord with most Logical Empiricists, such a moral stance is the result of a deeply personal and subjective decision. But this is not his goal anyway, since he doesn’t aim to change the epistemic and moral lives of his interlocutors; rather, the target of his methodological argument for realism are fellow empiricists—scientists and philosophers alike—who he expects to value testability; and the target of his axiological argument for testability are fellow humans who share the same humanist stance.

### Philosophical prescriptivism and the interventionist conception of philosophy of science

As detailed in the previous sections, around the late 1950s and early 1960s Feyerabend’s non-naturalist understanding of normativity provides a two-factor decision procedure: a methodological avenue, in which epistemic choices are guided, supported by considerations about means-ends relations and constrained by the notion of realizability; and an independent axiological avenue, in which a choice about epistemic values is made on the basis of moral values. However, realizability constraints became less and less important in Feyerabend’s reasoning over time, while the independent nature of the stipulation of epistemic values was put to the forefront.

An unpublished manuscript, “Notes on scientific method” from 1963, lays out Feyerabend’s notion of ‘methodology’ as it emerges from this non-naturalist construal of DBE:

[I]t is the purpose of methodology to design rules for the handling of statements which are supposed to constitute knowledge. Different sets of rules can be devised depending on different ideals of knowledge. The choice between these sets is a *genuine* choice which cannot be replaced by a stronger instance such as for example *proof*. Wherever proof occurs we are already moving *within* a chosen system and we have only failed to notice that a choice has been made. Nor does the *actual existence* of systems which have been built up in accordance with a certain set of rules give this set an advantage over other sets. There is method in many kinds of madness and what we want to find are methods we are able to approve of. Moreover the actually existing should not be exempt from criticism. Methodological discussions must not therefore be influenced by what does and what does not exist; and a methodologist must not be intimidated by the remark that his ideas “do not agree with”, say, “actual scientific practice”. Arguments between different methodologies may depend on demands not directly connected with the theory of knowledge. Thus the demand that the capabilities of human beings be developed as fully as possible, or the demand that a clear distinction be drawn between laws of nature and laws of society leads to the exclusion of certainty as an ideal of knowledge [...] (Feyerabend, “Notes on scientific method”, 4 February 1963^38^).

Feyerabend’s DBE, non-naturalistically construed, has a number of consequences for the relation between philosophy of science and science. It enables what I would like to call a *philosophical prescriptivism* *in methodological matters* because it reserves the right for philosophy to prescribe, quite independently of scientific practice, how science should proceed. Insofar as realizability concerns virtually vanish under this conception, this philosophical prescriptivism falls in the category of what Kaiser (2019, p. 43) has dubbed the “ex cathedra metanormativity” conception of philosophy of science, i.e. the view that “claims about a certain feature or element of science are not informed by and cannot fail in light of the empirical reality of scientific practice.”^39^ In 1963, Feyerabend also wrote a review of *Erkenntnislehre* by Viktor Kraft (1960), who had in the meantime abandoned his normative naturalist position and moved towards a non-naturalist conception of normativity much closer to Popper. In the review we read:

It is unfortunate that Professor Kraft does not follow up this excellent description of the [normative] *nature* of epistemology by an equally clear development of the *norms* adopted, of the *reasons* for the adoption of these norms, as well as a detailed argument showing how his position on various issues is influenced by these norms. This would have been a revolutionary undertaking indeed, the first construction of a purely normative epistemology (Feyerabend 1963, p. 320).

This prescriptivism in turn enables an interventionist conception of philosophy of science, according to which philosophy is neither in the business of merely reconstructing science and its (axiological) practice, nor in the business of aiding science from the perspective of the scientists’ stipulations. Rather, it is in the business of intervening in the development of science from the perspective of an independent axiological standpoint to advance human progress.

## Naturalist and non-naturalist conceptions of normativity in Kraft and Popper

In this section I want to give a historical explanation, with respect to the Feyerabendian context, of the two conceptions of normative methodology I introduced in the previous section, by showing that they can be traced back to the accounts of methodology of two protagonists of the voluntarist tradition of scientific philosophy, Viktor Kraft’s and Karl Popper’s. The former played a pivotal role in Feyerabend’s formative years, from 1948 to the early 1950s; the latter was to be Feyerabend’s mentor from the first half of the 1950s onwards. First, I show how naturalist and non-naturalist variants of the normativity of methodological rules can be traced back, respectively, to Kraft’s “bottom-up epistemology” (4.1) and Popper’s “theory of method” (4.2). I then argue that their accounts can be said to be quite similar in some respect and leading to different outcomes in another respect.^40^ In particular, the distinction between actual realization and conceivable realizability leads to a more “conservative” conception of axiological inquiry grounded in scientific practice, on Kraft’s side, and to a more “liberal” one, less grounded in scientific practices, on Popper’s side (4.3).

### Viktor Kraft’s ‘bottom-up epistemology’

Normative naturalism was not alien to Feyerabend already as a student. It is the conception that he learned from his teacher Viktor Kraft during his formative years in Vienna (Radler 2006; Kuby 2010).

In his outline of philosophy of science as a chapter of epistemology (*Wissenschaftslehre* or, more generally, *Erkenntnislehre*), Kraft criticizes the traditional way of handling epistemological problems:

[Traditional] epistemology proceeds almost thoroughly in a dogmatic way; it simply puts up its findings, it produces claim after claim without showing how they were attained. Its results are produced intuitively at best; their base remains untold (Kraft 1925, p. 3, my translation).

He counters the traditional way with another ideal of epistemology construed according to scientific philosophy (*Wissenschaftliche Philosophie*): “Epistemology has to establish its statements thoroughly like every other science” (Kraft 1925, p. 3, my translation). Kraft immediately notices that this puts epistemology on a par with its object of inquiry. Epistemology is not more fundamental than the sciences and thus cannot serve as a foundation for them. Instead, epistemology has to take up the sciences as the reference model of what knowledge actually is:

The endmost, which epistemology is all about, cannot be a proof that the sciences rightly claim to produce knowledge—that would suppose that we already have knowledge! Epistemology’s endmost aim is not a general justification of knowledge at all; its ultimate end is, rather, the actuality [Tatsächlichkeit] of knowledge. After all, what really establishes knowledge for us is not some dramatic event in our subjective experience, a self-awareness, a flash of insight, but rather the multiplicity of concrete scientific findings, which back up and support each other and become more and more productive (Kraft 1925, p. 6, my translation).

This sets out the first and most fundamental task of philosophy of science, the *descriptive task*:

Knowledge, as it exists concretely and actually in science, has to provide the factual basis for philosophy of science, from which the latter has to get off the ground and to which it has to refer back. The actuality [Tatsächlichkeit] of scientific knowledge is therefore the primary and unconditional premise of philosophy of science (Kraft 1925, p. 6, my translation).

Kraft has set out epistemology as a naturalized, anti-foundationalist enterprise. He calls it a “*bottom-up epistemology*, in contrast to the common ‘top-down’ approach”.^41^ But, in contrast to a common understanding of naturalism due to Quine, his naturalized theory of knowledge does not lose its grasp of the normative dimension of science. Similarly to Popper and Reichenbach, Kraft distinguishes between contexts of discovery and justification, but understands the distinction to be orthogonal to the normative-descriptive distinction.^42^ This allows for a descriptive study of the normative dimension of science. Kraft proposes a kind of normative naturalism, according to which “philosophy of science has to examine the single sciences with regard to their epistemological peculiarity, i.e. how they appear from the viewpoint of validity [Geltungsgesichtspunkt]” (Kraft 1925, p. 8, my translation). If philosophy of science learns from the sciences ‘what knowledge actually is’, it does so by learning about the sciences’ actual epistemic aims and values, their actual methods to achieve those aims, and their actual justification procedures. As we can see, the descriptive task views the sciences from the viewpoint of justification and is already axiological in character in that it deals with the validity claims of epistemic norms in science.

Furthermore, the descriptive task enables philosophy of science to carry out a second, *critical task*:

Knowledge, just as science, is a value [Wert], an ideal value, not just an actual one. And for this reason a critique of actual science is possible from the viewpoint of its axiological character [Wertcharakters], i.e. by testing to what extent it satisfies an ideal demand (Kraft 1925, p. 29, my translation).

In Kraft’s conception, the critical task of philosophy of science consists in holding the sciences accountable to their own ideal standards of inquiry.

The parasitic existence of philosophy upon the sciences gives a first answer to the questions how to choose aims: the choice of epistemic aims studied by philosophers of science mirrors the selection actually employed in the sciences. In a sense, scientific philosophers of science have no choice at all, they are bound to the axiological discourse in the sciences. However, philosophers can exploit the uneven development of scientific disciplines to help bring more stringent ideals into less rigorously developed parts of science (his early contribution was the establishment of geography as a proper science).^43^

Importantly, while Kraft’s value talk seems to set him apart from his fellow Vienna Circle members, his normative naturalism with regard to axiology does not imply any objectivist or (Neo-)Kantian notion of values. Epistemic values are, and remain, plain *stipulations* of actionable aims and thus defy epistemic means of justification (and what holds for ideals also holds for “every kind of rules or norms” (Kraft 1937, p. 168)).^44^ Science holds no special access to the true norms that should govern scientific research, indeed there is no matter of fact to which the ideal can conform. As Kraft puts it: “What is counted as science, is in principle just as arbitrary [*willkürlich*] and an issue of definition as in the case of artwork or morality” (Kraft 1925, p. 30, my translation)! Therefore, philosophy of science qua scientific philosophy may, in principle, stipulate epistemic ideals arbitrarily as well. However, in practice scientists do not pursue arbitrary epistemic goals. If philosophy of science sets its goal to be philosophy of the actual phenomenon called “science”, it has to take the successful axiology of science as a given. Ideals in science are thoroughly tested with regard to their feasibility and realizability; and the sciences, in their historical development, severely limit the infinite number of possible ideals down to those pursued successfully. These are the ideals that epistemology has to start from:

The ideal [...] is not set beforehand (“a priori”) with regard to actual science. [...] Instead, this ideal is construed in accord with actual science. The ideal of scientific knowledge ought to be developed starting from actual science. Only in this way the otherwise arbitrary stipulation can be determined. It makes no sense to set up an ideal without attending to the conditions of its realization” (Kraft 1925, p. 30, my translation).

This is a basic sketch of Kraft’s axiological conception. It is important to stress how this *normative* naturalism differs from blind acceptance of norms: Philosophy of science evaluates the reasons why such norms ought to be accepted and epistemic norms in science are taken for granted because a successful warranting process has taken place according to these standards. (The fact that the standards to evaluate the epistemic norms of science are taken over from science may be anathema to a foundationalist conception of knowledge, but is at the core of the bootstrapping process of Kraft’s anti-foundationalist conception.) Kraft’s axiological conception delivers an answer to the question about what can guide philosophy of science in its choice of epistemic aims (the axiology of science) and what can justify this choice (the reasons according to which scientists select the norms of science).

Kraft’s setup of philosophy of science, its descriptive and normative task, is captured in Feyerabend’s two-pronged arguments for methodological rules: The normative argument has to present methodological rules as hypothetical imperatives connected to epistemic aims; the descriptive argument has to provide evidence that those aims are realizable—the most straightforward way being that they actually have been realized in scientific practice. (Feyerabend’s negative arguments against methodological rules show the converse: the methodological rules under scrutiny hinder some epistemic aim cherished by the argumentative opponent; and the rules under consideration were not followed as a matter of fact by the scientists which pursued the very same epistemic aims.^45^)

### Karl Popper and descriptive function of axiological stipulations revisited

Historically, the idea of scientific philosophy did not produce a unified conception of what it means to do philosophy. The concept of scientific philosophy is relative to which science—*Wissenschaft*, thus not limited to the natural sciences—is taken as a reference point. Kraft’s view of science was heavily informed by the concept of “theory” found in mathematical physics. Accordingly, he conceived of the general concept of scientific theory as a deductively structured system of sentences. His appreciation of deduction led him to present deductive inference both as a central feature of justification *and* of discovery in the sciences, leading him to propose a general hypothetical-deductive account of scientific theory in his (1925), a view more generally associated with Karl Popper’s development of falsificationism. Contrary to Popper, Kraft never denied the (limited) role of inductive reasoning either in theory formation nor as a (fallible) mean of theoretical justification. Indeed, he calls his two-step setup of philosophy of science “critical induction” (Kraft 1925, pp. 28–31), recognizing the basic role that piece-meal inductive generalization plays in the bootstrapping process.^46^

Kraft’s setup has one important outcome regarding the notion of realizability and, consequently, the scope of philosophy of science. While in the previous methodological section the notion of realizability applied to an indefinite amount of possible aims that may be realized, “critical induction” reduces the notion to a limited set of aims which actually have been realized in science. This not only resolves the problem of choice, but it also sets a general *conservative axiological attitude* of philosophy of science.

Contrast and compare this result with Popper’s similar descriptive demand of philosophy of science in his posthumously reconstructed *Die beiden Grundprobleme der Erkenntnistheorie* (1930–1933), his first try at the philosophical views which would become famous in his *Logik der Forschung* (Logic of Scientific Discovery, (1934)). In his early notes, Popper introduces the notion of a “transcendental method” (*transzendentale Methode*) of epistemology demanding an “orientation towards the actual scientific activity” (Popper 2010, p. 152, my translation):

The results of the *theory of method* [*Methodentheorie*] are to be compared with the actually successfully employed methods of the empirical sciences. Theories of knowledge which fail to achieve a satisfactory representation of the actual methodological procedure have to be regarded—and therein consists the transcendental method—as failed (Popper 2010, p. 548, my translation).

Popper sets up a descriptive aim that philosophy of science has to fulfill, too. But this condition is conceived as a *deductive test* of philosophy of science with regard to science (as one would expect, given Popper’s conception of scientific method). According to Popper, all philosophical accounts of science are in a “transcendental competition” to account for “actual procedures of justification in science” (Popper 2010, p. 548, my translation).

### The scope of axiological inquiry: actual realization or conceivable realizability?

I propose to capture the difference between Kraft and Popper as a distinction between (1) inductively-gained axiological stipulations; and (2) axiological stipulations that are put to descriptive test. Popper’s conception allows us to put the stipulative nature of methodological rules at the forefront, stressing their a priori character. For this reason Popper’s conception has been called a “normative apriorism”, ascribing to him the thesis that “scientific method is a matter of a priori reflection and justification” (Rosenberg 1990, p. 34). In light of the descriptive pretension highlighted in Popper’s *Die beiden Grundprobleme* I think this ascription to be inadequate. This is not to say that there’s no disagreement between the two conceptions: Popper’s characterization of methodological rules allows to sharply separate the stipulative nature from the fact-sensitive (descriptive) pretension of axiological considerations—and this conceptual division is no accident. One of Popper’s targets of his sharp distinction between “nature” and “convention” is a naturalist solution to the lack of axiological foundation and this distinction is seemingly violated in a naturalist conception of normativity. Popper (1959, p. 31) explicitly mentions Kraft (1925) as a vicious example of naturalism (thereby distorting Kraft’s proposal by neglecting its normative dimension). From the viewpoint of normative naturalism, on the other hand, Popper’s conception of methodological rules is seen as giving up on the question of axiological deliberation, giving in to arbitrariness.^47^

Both accusations miss their target. On the one side, Kraft’s normative naturalism is anti-foundationalist, too. A descriptive survey of scientific axiological stipulations doesn’t make their stipulative nature disappear. Kraft distinguishes between the stipulative nature and the fact-sensitive pretension of axiological considerations as well. On the other side, Popper’s highlighting of the stipulative nature of methodological rules is a move against foundationalist solutions generally, including naturalist ones, not a claim of the rules’ (unsurmountable) arbitrariness. After all, the descriptive constraint is not dropped, but is postponed, encoded in the “transcendental method” as a further step. The outcome is quite similar: Both Kraft (1925) and Popper (2010) set the task of philosophy of science to account for actual scientific methodological and axiological practice.^48^

The difference between inductively-gained axiological stipulations and axiological stipulations that are put to descriptive test shows itself elsewhere, in that it has a major consequence for the notion of realizability: actual realization is traded in for conceivable realizability. The methodological rules considered in the setup (as opposed to the validation) of Popper’s *Methodentheorie* are, in principle, all conceivable aims that may be realized, not the limited set of aims which actually have been realized in science. This has serious implications for the scope of philosophy of science, in that it positions philosophy in the business of investigating the realm of possible epistemic aims, instead of restricting it to actual epistemic aims used in the sciences. This difference translates to a more liberal attitude with respect to axiological inquiry: the setup of axiology in philosophy of science is divorced from a descriptive task, it is not limited by the axiology of actual science.^49^ The axiology of actual science is only introduced as a further step to certify or decertify axiological stipulations.

The normative naturalist’s protest has a point though: Insofar as the stipulation of rules is practically dissociated from their descriptive function, the latter is in need of a rationale of its own—why should axiological stipulations in philosophy of science be at all certified by axiological stipulations found in actual science? As if Popper wanted to confirm this worry, the descriptive test is nowhere to be found as an explicit requirement in his later *Logic of Scientific Discovery* (1934). Here two sources of deliberation, stipulation and realizability, which are inextricably linked in the naturalist conception, are permitted to go separate ways.

The consequences of this latter possibility we can see best in Feyerabend’s philosophical prescriptivism about axiology in science (presented in Sect. 3.4): realizability concerns almost vanish; methodological demands cannot fail in principle in light of the axiological practice of science; and philosophy of science acquires standing to intervene in the development of science from an independent axiological vantage point.

In summary, we can take stock of two variants in the conception of normativity within the voluntarist tradition of philosophy of science, exemplified by Kraft and Popper. At various times during his intellectual life, Feyerabend adopted either a naturalist or a non-naturalist conception of normativity. During his formative years in Vienna, he followed Kraft’s early naturalist conception of normativity; then, under Popper’s philosophical guidance, he came to defend a strong non-naturalist conception; and, finally, around the mid 1960s, Feyerabend adopted a conscious naturalist conception of normativity by developing a more refined account of how methodological rules are embedded in scientific practices.

## Conclusion

In this paper I give an account of Feyerabend’s metaphilosophy in terms of Decision-Based Epistemology. My goal was to identify key conceptual resources present in his metaphilosophy, such as epistemic voluntarism, a behaviorist strategy, and the non-naturalist and naturalist variants of his conception of normativity, which he applied in the context of methodology. The former variant, peaking in the first part of the 1960s, enabled an independent axiological justification of epistemic values on the basis of moral values and it equipped general philosophy of science with an interventionist task towards the sciences. The latter variant, from the mid 1960s onwards, collapsed axiological justification of scientific values back into methodology, leading to the proposition that such justification process can only occur in the contextual research situation.

This development, however, does not imply a complete abandonment of DBE. I promised a framework in which major philosophical changes in Feyerabend’s historical turn could be accounted for by more refined changes in his metaphilosophy. Indeed, the only change concerns the way in which the epistemic aim in arguments about means-end relations regarding scientific behavior is stipulated: instead of giving in-principle reasons derived from ethical concerns, the reasons can only be given contextually in scientific deliberation. This has the consequence of making epistemic demands in science not decidable on the ground of general axiological reasoning, thus making Feyerabend’s view of general methodology obsolete; but this does not imply that axiological reasoning is therefore obsolete, too. One of the surprising continuities with Feyerabend’s later thought is that, while DBE’s axiological reasoning cannot justify philosophical prescriptivism with regard to science anymore, it continues to play an important role in his social philosophy and epistemology. He would eventually apply axiological holism and consistency considerations to the notions of “forms of life” and “traditions”. His strong thesis of an entailment between moral and epistemic values would be weakened insofar as the relation turns out to be underdetermined, but would continue to be sustained within a much broader concept of knowledge. As late as 1992, two years before his untimely death, Feyerabend would maintain that the very fact that scientists existentially quantify over something is dependent on moral values:

The predicate “real,” [...] is only apparently descriptive. Reflecting a preference for forms of coherence that can be managed without too much effort, it contains evaluations, though implicit ones. Now wherever there is a preference there can be, and perhaps should be, a counterpreference. For example, we may emphasize human freedom over easy manageability. This means, of course, that ethics (in the general sense of a discipline that guides our choices between forms of life) affects ontology (Feyerabend 1992, p. 111).

Feyerabend’s DBE lasted till the end.

There are many outstanding questions regarding DBE as a framework for reconstructing Feyerabend’s metaphilosophy. As the three main components presented in Sect. 2 about how one can and ought do philosophy (of science) are all but obvious, a proper reconstruction of Feyerabend’s DBE involves at least three tasks: (1) to give an account of just what Feyerabend’s beliefs are; (2) to reconstruct how these beliefs came about; and (3) to examine possible and historically plausible justifications for these beliefs. In this article I have expanded on (1) and gave some indications to answer (2), but remained almost silent with regard to (3). As briefly indicated at the beginning of Sect. 2, I think that the latter task can be tackled by framing Feyerabend’s metaphilosophy in a much larger voluntarist tradition within scientific philosophy, in which a variety of arguments for various forms of epistemic voluntarism were first developed and on which Feyerabend’s metaphilosophical position tacitly rests.

## Footnotes

1 For the notion of *stance*, see footnote 13, below. A more detailed discussion about the difference between the practice of philosophy and its metatheory would go beyond the scope of this paper. The term ‘metaphilosophy’ is by now an established term, but is still to be used with caution, as Williamson notes when opting for the alternative term ‘philosophy of philosophy’: “I [...] rejected the word ‘metaphilosophy’. The philosophy of philosophy is automatically part of philosophy, just as the philosophy of anything else is, whereas metaphilosophy sounds as though it might try to look down on philosophy from above, or beyond” (Williamson 2007, p. ix). I agree with Williamson’s understanding of the subject matter. (I would add that a peculiarity of philosophy is the ongoing renegotiation of its own metaphilosophical perspective.) In using “metaphilosophy” I certainly do not mean to imply Williamson’s worrisome suspicion. All the elements of Feyerabend’s metaphilosophy are indeed elements discussed in philosophical branches of epistemology, action theory and ethics.

2 Preston (1997) does not explicitly distinguish between Feyerabend’s philosophical arguments and his metaphilosophy. However, Preston’s ascription of a “philosophical project” to the early Feyerabend includes *en bloc* both a reconstruction of the philosophical claims which Feyerabend defended as well as the “normative” (Preston 1997, pp. 14, 16–17, 20–22, 178) conception of philosophy from which Feyerabend is said to have argued for his claims, which is plausibly a reconstruction of his early metaphilosophical conception. Preston’s diagnosis that the later Feyerabend emerged by “losing interest in the ambitions of this earlier project” (Preston 1997, p. 8) comes from his “belated recognition of the poverty of the normative approach to philosophy of science” (Preston 1997, pp. 178–179], thus not only relinquishing his previous philosophical claims, like methodological monism, but also abandoning the normative conception and adopting a new one, which Preston variously labeled as a “descriptive” (Preston 1997, p. 14), “historical” (Preston 1997, pp. 7, 83) or “naturalistic” (Preston 1997, pp. 14, 110) conception of philosophy of science.

3 Among others, I subsume diverse philosophers such as Rudolf Carnap, Otto Neurath, Philipp Frank, Hans Reichenbach, Karl Popper and Hugo Dingler under this heading. William James is also a voluntarist scientific philosopher in this sense. Furthermore, the roots of this tradition may be traced back to the geometric and physical conventionalism of Henri Poincaré and Pierre Duhem in France and the *Lebensphilosophie* of Friedrich Nietzsche in Germany. Bas van Fraassen, the most noteworthy contemporary proponent of epistemic voluntarism in philosophy of science, latches onto the voluntarism in the tradition of existentialism.

4 Closely connected is Feyerabend’s early critique of the “historical turn” in philosophy of science in the late 1950s/early 1960s and his subsequent change of mind. See Kuby (forthcoming) for a reconstruction of these changes.

5 In this respect I agree with Preston (1997, p. 20)’s assessment that “one of the most consistent themes in [Feyerabend’s] work as a whole is his strong voluntarism”. Unfortunately, Preston does not follow up on this insight; indeed, he never engages with “voluntarism” in the course of his book. The exception to the rule can be found in the closing sentences of a subchapter: “Voluntarism is, as I noted in chapter 1, a very strong theme throughout Feyerabend’s thought. It chimes with his insistence that ethical decisions dictate at least the form of human knowledge” (Preston 1997, pp. 195–196).

6 The modern discussion started with the James-Clifford debate on the possibility of an “ethics of belief”. Since then the discussion has been extensive, see Chignell (2016) for an overview of the literature.

7 The strong variant called direct doxastic voluntarism, which would be implied here, is deemed implausible because it is widely assumed, for empirical and conceptual reasons, that belief-formation is not a voluntary act—we cannot believe propositions at will—and therefore belief-formation is not sensitive to pragmatic reasons in the way in which e.g. actions are.

8 For an account of the relationship between Logical Empiricism’s use of behaviorism and psychology, see Smith (1986). A defense of the sophisticated kind of “logical behaviorism” is given by Kaila (2014, Ch. 9) and Crawford (2013) defends logical behaviorism against similar misinterpretations.

9 Carnap’s pragmatic theory of observation, modeling observation sentences as trained linguistic reactions of an agent to some event in her surroundings, is a prime example (see Kuby 2018).

10 Though quite radical in its deflationary outcome, we will want to ascribe to Feyerabend a minimal version of this strategy: it is a non-reductive account of belief, i.e. Feyerabend never implied that beliefs should be reduced to such behavioristically interpreted mental states; it is also not intended as a general account of belief, i.e. Feyerabend never implied that all and every belief can or should be interpreted this way.

11 See Psillos (1999) for the standard account of the three-components model of scientific realism, comprising ontological, semantic and epistemological claims.

12 See for example Feyerabend (1954, esp. 474–475, 1958a; b; 1960b, esp. 157–159, 1964).

13 Though they may *include* beliefs. While less radical, a first approximation to Feyerabend’s understanding of a philosophical position, including realism, is perhaps van Fraassen’s notion of a philosophical “stance”. Van Fraassen contrasts the notion of stance to that of a “doctrine”, a body of truth-apt claims, in that a stance comprises “attitudes, commitments, values, goals” (van Fraassen 2002, p. 48) and is thus sensitive to further pragmatic considerations.

14 See e.g. Feyerabend’s retrospective account of his earlier view in his last interview (Jung 2000, pp. 161–162): “[Interviewer]: What does rationality mean for you?” “F[eyerabend]: A set of rules which you are supposed to follow and which says: ‘If so then it will be this and that’, for example: ‘Always avoid contradictions’ ”.

15 The normative dimension of Feyerabend’s early philosophy of science has been highlighted by many Feyerabend scholars, see Couvalis (1989, p. 132), Preston (1997, pp. 14–17), Farrell (2003, pp. 147–151), Oberheim (2006, pp. 88–92).

16 Laudan explicitly argues “that *all* methodological rules (at least all of those rules and constraints of the sort usually debated among methodologists) can be re-cast as contingent connections between ends and means” (Laudan 1987, p. 25).

17 Note that the deontic modality of prescription (“should” or “ought”) in (1) is only—strictly speaking—warranted if there are no other means *G* to achieve *N*. Laudan circumvents the problem by making the validity of (1) hinge on the reliableness of a statistical law of the form “Doing *G* is more likely than its alternatives to bring about *N*” (cf. Laudan 1987, p. 25). But, contrary to Laudan’s presumptive view, note that (2) alone doesn’t justify (1). There is no compulsion to do *G*_1_ if another action *G*_2_—even if less likely—still produces *N*. A further step is needed, which extends the notion of realizability as limited constraint and adds ‘maximized utility’ considerations—for maximizing one’s chances to succeed is surely stronger a demand than the avoidance of self-sabotage by aiming at unrealizable goals. We might want to charitably include such considerations into the “obvious riders and qualifications”, which Laudan assumes “are needed to establish the warrant-conditional substitutivity of (2) for (1)” (Laudan 1987, p. 30, fn. 18).

18 This point is also forcefully argued by Laudan (1987, pp. 26–27).

19 Timothy Lyons (2005) has argued against this requirement, as found in Laudan, to defend “purely axiological realism”: One argument for why it is not irrational for science to seek true theories, even if most theories in the history of science have been falsified, is that it is not irrational in some circumstances to pursue “epistemically utopian goals” (Lyons 2005, p. 168), i.e. epistemic aims which we have good reason to believe are unattainable. An argument for the latter claim, building on Kant, has been put forward by Rescher (1982, p. 227).

20 Testability is but one out of a tightly related family of epistemic values from which Feyerabend argues for realism. See footnote 12 for exemplary references to Feyerabend’s writings arguing for realism.

21 But less and less so since Psillos (2011) has developed sympathies for realism as a framework sensitive to pragmatic considerations, taking his cue from Van Fraassen’s stance empiricism (van Fraassen 2002); see also the contributions in Forbes (2018). Chakravartty (2017) similarly defends a voluntarist approach to scientific ontology distinguishing a range of “empiricist” “metaphysical stances”.

22 It is for this reason that van Fraassen’s constructive empiricism is considered not to be a kind of reductive empiricist account. Semantic realism has become common ground in the debate, cf. *inter alia* Psillos (2009, p. 5): “Semantic realism is not contested any more. Theoretical discourse is taken to be irreducible and assertoric (contentful) by all sides of the debate.” See further Oberheim (2006, pp. 180–191) for a good discussion of the differences between scientific realism and Feyerabend’s methodological realism.

23 See Neuber (2015) for an exhaustive account of realist claims in Logical Empiricism.

24 Feyerabend’s case may be more complicated still. I can only hint at the fact that much of the problems encountered in classifying Feyerabend’s self-professed “realism” on the spectrum of the contemporary realism–anti-realism debate are quite similar to Quine’s self-professed “robust realism”.

25 Feyerabend (2015, pp. 22–23, amended translation; cf. Feyerabend 1954, pp. 474–475): “Most theories, world-views of past physics turned up to be illusions. Who ensures us that we are not falling victim to an illusion again in the present? Answer: No one can give us such a warrant. But does it follow from this that we should formulate our theories in a more cautious manner from now on [i.e. in a ‘positivist’ manner]? Such a course of action would make it impossible to carry out the scientist’s most important task: to correct scientific theories. Who doesn’t assert anything, cannot be refuted. [...] Hence we must use our theories as though we were completely convinced of their trueness, we must speak as though the world was really constituted in the manner asserted by the theory [...]. Briefly put: We have to conceive of physical theories as they prima facie present themselves—as statements about the world. What is forbidden to a physicist—to surround his theories with cautionary clauses—seems to be permitted, indeed imperative to a philosopher; the latter recognizes that the physicist’s statements are not necessarily valid, that they have hypothetical character”.

26 Note that a *normative* naturalist does not simply appeal to values as facts; by referencing the axiological practice of science, she does consider the axiological choice to be justified: values are tested and discarded or corroborated. Still, the standards of justification which warrant the axiological choice are those of science; here normative naturalism displays the characteristic bootstrapping structure of *naturalism*.

27 I thank Martin Kusch for bringing to my attention this more ‘descriptivist’ interpretation of the passage.

28 A book Feyerabend was intimately acquainted with, having produced the first German translation of it (Popper 1957). He references Chapter V as a source convincingly arguing the shortcomings of naturalism in Feyerabend (1958a, p. 147) and Feyerabend (1960c, p. 336).

29 This is a direct application of Popper (1959, p. 30): “I reject the naturalistic view [of methodology]. It is uncritical. Its upholders fail to notice that *whenever they believe themselves to have discovered a fact, they have only proposed a convention.* Hence the convention is liable to turn into a dogma. This criticism of the naturalistic view applies not only to the [positivist] criterion of meaning, but also to its idea of science, and consequently to its idea of empirical method.”

30 See Kuby (in preparation), for an interpretation to understand this debate.

31 For interpretations of Feyerabend’s later philosophy, see Preston (1998), Farrell (2001), Kidd (2012), Kidd (2016), Tambolo (2014), Martin (2016), Heller (2016), Sorgner (2016), Kusch (2016), Brown (2016) and Shaw (2017).

32 This thread has been highlighted by Farrell (2003, p. 150) under the heading of “axiological normativity” and expanded into a perspicuous account of Feyerabend’s overall philosophy in the same book.

33 Kuby (in preparation) offers a critique of Feyerabend’s conception of “partisan” historiography he defended against Kuhn.

34 How far reaching Feyerabend thinks these interrelations are, is well exemplified by his remark that the “choice of the critical form of life will have repercussions in very distant fields, for example, in the dramatic arts” (Feyerabend 1962, p. 71).

35 Cf. Feyerabend (1965, p. 146) for an even stronger pronouncement: “The point made in the text and slightly elaborated here would seem to imply that the problem of responsible choice enters even the most abstract philosophical matters and that ethics is, therefore, the basis of everything else.”

36 Reichenbach (1961, p. 13): “The system of knowledge is interconnected in such a way that some decisions are bound together; one decision, then, involves another, and, though we are free in choosing the first one, we are no longer free with respect to those following. We shall call the group of decisions involved by one decision its *entailed decisions*.”

37 The ethical foundation of Feyerabend’s (meta)philosophy has been worked out early on by Hooker (1972, pp. 490–497). While there are important parallels with the connection that Popper’s critical rationalism draws between epistemology and social philosophy, the degree of agreement between Feyerabend and Popper depends on an interpretation of the latter’s overall philosophy. Without doubt both agree on the moral nature of the decision for the epistemic values of criticism (“The choice before us is not simply an intellectual affair, or a matter of taste. It is a moral decision. [...] For the question whether we adopt some more or less radical form of irrationalism, or whether we adopt that minimum concession to irrationalism which I have termed”critical rationalism“, will deeply affect our whole attitude towards other men, and towards the problem of social life” (Popper 2012, II:232).) Both also agree that social and political conditions are necessary to realize the epistemic value of criticism. But, according to Feyerabend, in Popper’s case ethical concerns follow from the epistemic concerns, not the other way around. It is the scientific method that encapsulates the cherished epistemic virtues of criticism. Critical rationalism is a generalization of those virtues to society as a whole. Opposed to this, in Feyerabend’s conception moral values are primary—and it so happens that the scientific enterprise embodies an exemplary realization of the epistemic values entailed by a specific moral attitude. Feyerabend makes this point when arguing his preference for the philosophy of Mill over Popper’s: “The difference between Mill and Popper, however, seems to me to lie in this. For Mill the (material and spiritual) welfare of the individual, the full development of his capabilities, is the primary aim. The fact that the methods used for achieving this aim also yield a scientific philosophy, a book of rules concerning the ‘search of truth’, is a side effect, though a pleasant one. For Popper the search for the truth seems to be much more important and it seems occasionally to even outrank the interests of the individual. In this issue my sympathies are firmly with Mill” (Feyerabend 1970, p. 110, fn. 48). To be sure, Feyerabend’s pushed this interpretation of Popper’s philosophy only *after* breaking up with critical rationalism in public. And this reading is at odds with the common interpretation, which puts Popper much more in agreement with Feyerabend (see Gattei 2002, for an overview of key literature). Collodel (2016) delivers a masterful account to understand Feyerabend’s early self-fashioning as a “Popperian” and its eventual abandonment.

38 HF 08-34-05, Herbert Feigl Papers, Archive for Scientific Philosophy, Hillman Library, University of Pittsburgh.

39 In this respect I disagree with Oberheim (2006, pp. 88–92)’s contention that Feyerabend’s use of arguments involving descriptive adequacy with respect to scientific practice *before 1964* “can be properly placed within the historical school” (90) and could therefore serve as evidence against my claim. For an argument, see Kuby (in preparation).

40 See Radler (2018) for a wider, historically-grounded philosophical comparison between Kraft and Popper.

41 Kraft (1925, p. 10): “*eine Erkenntnistheorie ‘von unten’* gegenüber der ‘von oben’, wie sie üblich ist”.

42 The former (discovery-justification) are viewpoints from which we can assess our objects of inquiry: are we interested—Kraft uses the older Neo-Kantian terminology—in an account of their “formation” (*Genese*) or an account of their “validity” (*Geltung*)? The latter (normative-descriptive) are tasks of philosophy which we can set.

43 See Kraft (1914, 1929). Kraft received training in geography under Albrecht Penck, a specialist in physical geography and geomorphology. Penck was influenced by William Morris Davis, who first delivered a deductive theory of geomorphological change (see Radler 2012, §.c)

44 For an overview of the incredibly varied picture of axiology, morals and ethics in Logical Empiricism, see Siegetsleitner (2010, 2014).

45 Oberheim (2006, p. 51) similarly highlights Feyerabend’s “two-pronged argumentative strategy”, but without noticing the Kraftian heritage, explaining that “such two-pronged attacks argue historically (or descriptively) that some philosophical thesis does not match actual scientific practice, and methodologically (or normatively) that the thesis would have undesirable consequences”.

46 The “inductive” part refers to the piece-meal survey of all the sciences; the “critical” part to a ranking of the sciences according to the rigor of their methods, preferring stricter (yet realizable) aims to more easily achievable ones.

47 Laudan (1987, p. 19) positions his normative naturalism against Popper, chastising his conception of methodological rules: “Popper generally treats methodological rules as conventions, between which no rational choice can be made”. Kraft voices a similar sentiment, targeting previous Neo-Kantian accounts of science: “[A]ll too often epistemological statements turn out to be mere constructs without any relationship to real knowledge. This turns out to be all too easy, as epistemology roams freely in a world of abstract generalities, almost without no tying in with concrete knowledge, and even then only to give singular examples and references” (Kraft 1925, p. 29, my translation).

48 The substantive philosophical matter of contention concerns the question: are there a priori, analytic methodological rules? Are the kind of rules proposed by the early Popper meant to be of this kind?

49 As an anonymous reviewer noted, feminist approaches to philosophy of science, like Helen Longino (1995)’s discussion and defense of alternative values in science (novelty, ontological heterogeneity, complexity of interaction, applicability to human needs, diffusion of power), have built tacitly on this liberal approach to axiology in science.
